# Electrochemical skin conductance to detect sudomotor dysfunction, peripheral neuropathy and the risk of foot ulceration among Saudi patients with diabetes mellitus

**DOI:** 10.1186/s40200-016-0252-8

**Published:** 2016-08-05

**Authors:** Eman Sheshah, Amal Madanat, Fahad Al-Greesheh, Dalal AL-Qaisi, Mohammad AL-Harbi, Reem Aman, Abdul Aziz AL-Ghamdi, Khaled AL-Madani

**Affiliations:** Diabetes Care Center, King Salman Hospital (MOH), Ayesha Bint Abi Baker St., PO Box: 15169, Riyadh, 11444 Kingdom of Saudi Arabia

**Keywords:** Sudomotor dysfunction, Sudoscan, Diabetic peripheral neuropathy, Neuropathy disability score

## Abstract

**Background:**

Sudomotor dysfunction is manifested clinically as abnormal sweating leading to dryness of feet skin and increased risk of foot ulceration. The aim of this study was to test the performance of foot electrochemical skin conductance (ESC) to detect diabetic peripheral neuropathy and the risk of foot ulceration against traditional methods in Saudi patients with diabetes mellitus.

**Methods:**

This cross-sectional study was conducted on 296 Saudi patients with diabetes mellitus. Painful neuropathic symptoms were evaluated using the neuropathy symptom score (NSS). The risk of foot ulceration and diabetic peripheral neuropathy were determined using the neuropathy disability score (NDS). Vibration perception threshold (VPT) was assessed using neurothesiometer. Neurophysiological assessment of the right and left sural, peroneal and tibial nerves was performed in 222 participants. Diabetic peripheral neuropathy was defined according to the definition of the American Academy of Neurology. ESC was measured with Sudoscan.

**Results:**

Feet-ESC decreased as the scores of sensory and motor function tests increased. Feet-ESC decreased as the NSS, NDS and severity of diabetic peripheral neuropathy increased. Sensitivity of feet-ESC < 50μS to detect diabetic peripheral neuropathy assessed by VPT ≥ 25 V, NDS ≥ 3, NDS ≥ 6 was 90.1, 61 and 63.8 % respectively and specificity 77, 85 and 81.9 % respectively. Sensitivity of feet-ESC < 70μS to detect diabetic peripheral neuropathy assessed by VPT ≥ 25 V, NDS ≥ 3, NDS ≥ 6 was 100, 80.6 and 80.9 % respectively. Sensitivity and specificity of feet-ESC < 70μS to detect confirmed-diabetic peripheral neuropathy were 67.5 and 58.9 % respectively.

**Conclusion:**

Sudoscan a simple and objective tool can be used to detect diabetic peripheral neuropathy and the risk of foot ulceration among patients with diabetes mellitus. Prospective studies to confirm our results are warranted.

## Background

Diabetic autonomic neuropathy is a disorder of the autonomic nervous system affecting the cardiovascular, gastrointestinal and urogenital systems and sudomotor function in the setting of diabetes mellitus (DM) and metabolic derangements of pre-diabetes after exclusion of other causes [1].

In the context of diabetic peripheral neuropathy (DPN) sudomotor dysfunction (SMD) can occur in two phenotypes: either as one component of a generalized DPN, or concurrently in distal small fiber sensory polyneuropathy (SFSN) [2].

Sudomotor C-fibers are postganglionic, unmyelinated, cholinergic, sympathetic nerves that innervate sweat glands, and SMD is manifested clinically as abnormal sweating leading to skin dryness. Dryness of foot skin as a result of SMD is associated increased risk of foot ulceration (FU) [3].

Prevention of FU in patients with DM continues to represent an important issue, as the steady rise in the prevalence of DM is leading to a persistent burden of DM-related complications including lower limb amputations [4]. Approximately 84 % of non-traumatic amputations occurring in diabetes are preceded by FU [5]. While peripheral artery disease accounts for an increased risk of FU in only 35 % of cases, DPN contributes to 78 % of the risk of FU, and together with foot deformity and repetitive trauma forms the clinical triad that leads to FU [5, 6].

As such, every effort is needed to detect DPN including SMD early in the course of the disease in order to identify those patients in need of special care to minimize the risk of FU and prevent limb loss. Utilization of tests to assess SMD in daily practice has been limited, as the known standardized methods to do so are either invasive, complex, time-consuming or require specialized equipment and training [2].

Recent introduction of sudorimetry technology using Sudoscan which measures electrochemical skin conductance (ESC) of the hands and feet based on reverse iontophoresis and chronoamperometry has allowed rapid, noninvasive, robust, accurate assessment of sudomotor and small nerve fiber function [7]. ESC measurement requires little technical training and no calculations and can easily be integrated into daily practice.

Over the past three decades the prevalence of DM in Saudi Arabia has increased approximately 10-fold [8]. As DPN is the earliest and most common long term complication of DM [5, 9], it is imperative to validate and utilize novel screening and diagnostic methods [10], in particular those that are simple, non-invasive, easy to use at a point of care and that have the potential to overcome barriers to screening for and detecting DPN including SMD.

The aim of our study was to test the performance of feet-ESC to detect DPN and the risk of FU against traditional methods among Saudi patients with DM.

## Methods

A cross-sectional observational study was conducted at the diabetes care center, King Salman hospital in Riyadh, Kingdom of Saudi Arabia between January and May 2012. Informed consent was obtained from all participants.

### Study population

Participants in the study consisted of two hundred ninety six Saudi Arabian patients with DM referred for the first time from primary care health centers for general diabetes care. Exclusion criteria included patients younger than 18- or older than 65 years of age; patients with secondary causes of DPN, peripheral vascular disease or active foot ulcer; or patients taking drugs that affect autonomic function testing such as β-blockers or atropine.

### Clinical assessment

Physical examination including detailed feet assessment was conducted by physicians as described by Boulton AJ et al [11]. Characteristics of the appearance of the feet were documented for the presence of dryness, fissures, calluses and deformities.

Painful neuropathic symptoms (PNS) were evaluated using the neuropathy symptom score (NSS) [12]. Symptoms were considered positive if NSS ≥ 5. Diabetic peripheral neuropathy and the risk of FU were determined using the neuropathy disability score (NDS). DPN was present if NDS ≥ 3. Risk of FU was present if NDS ≥ 6 [12]. Severity of DPN was graded according to NDS scores: None (0–2), Mild (3–5), Moderate (6–8), Severe (9–10) [12]. Scores for NDS were derived from ankle reflex testing using a reflex hammer, vibration sensation using a 128Hz tuning fork, pain sensitivity using the neurotip and differences in temperature sensation using warm cold rod. Pressure sensation was tested using a 10-g monofilament as described by Boulton AJ et al [11]. Quantitative sensory testing (QST) to determine vibration perception threshold (VPT) was performed using a neurothesiometer on both halluces. Complete blood count was performed using (Hematology Analyzer, Sysmex XT 2000i), HbA1c was performed using (Dimension Xpand™ Rxl max, Hemoglobin A1c), the method has been certified for precision and accuracy by the National Glycohemoglobin Standardization Program (NGSP) ensuring clinical results consistent with the findings of the Diabetes Control and Complication Trial (DCCT). Blood chemistry was performed in the morning after 12 h fasting using (Chemistry Analyzer, Dimension Xpand™ Rxl max).

### Neurophysiological assessment

Nerve conduction study was performed in 222 participants using Nicolet Viking Quest, VIASYS Healthcare Inc Neurocare Group USA. Right and left sural sensory amplitude and conduction velocity and peroneal and tibial motor amplitudes, latencies and conduction velocities were measured. Results were compared to age specific normal values to determine nerve conduction study abnormality. Participants were then classified as having normal, subclinical (ie nerve conduction abnormality but without symptoms or signs of DPN) or confirmed DPN as described by Tesfaye S et al 2010 [1]. Confirmed DPN was present if the participant had at least one symptom or sign of DPN and one or more abnormal nerve conduction test in both sural (sensory) and peroneal or tibial (motor) nerves in accordance with the case definition of DPN described by the American Academy of Neurology [13].

### Sudomotor assessment

Measurement of ESC of both hands and feet was performed using Sudoscan. Participants placed their hands and feet on two sets of large-stainless-steel electrodes which were connected to a computer for recording and data management. Electrochemical skin conductance is the ratio of the current measured over the constant power applied expressed in micro-Simens (μS) for the hands and feet (right and left sides). Through reverse iontophoresis, the device generates voltage to the cathode and a current (intensity of around 0.2 mA) occur between the anode and cathode proportional to sweat chloride concentration. At low voltage (<10 V) the stratum corneum is electrically insulating and only sweat gland ducts are conductive. Sudomotor dysfunction is absent if measured hands-ESC is ≥60μS or feet-ESC ≥ 70μS. Moderate SMD is present if measured hands-ESC is ≥40 but < 60 μS or feet-ESC is ≥50 but < 70 μS. Severe SMD is present if measured hands-ESC is <40μS or feet-ESC is <50μS. These thresholds have been defined based on previous studies [14–16]. The test lasts less than 3 min and is painless.

### Statistical analysis

Data are presented as mean ± SD or percentages. Pearson’s correlation coefficient was used to evaluate the relationship between feet-ESC and clinical and biochemical variables as well as bedside-tests, QST, and neurophysiologic testing to detect DPN. Variances between variables were calculated using an independent *T* test. For all the tests a P value of 0.05 or less was used for statistical significance. Sensitivity and specificity for feet-ESC to detect DPN and the risk of FU were calculated against the cutoff values of VPT ≥ 25 V, NDS ≥ 3, confirmed-DPN for the presence of DPN and NDS ≥ 6 for the presence of increased risk of FU using Bland-Altman plots [12, 17, 18]. Receiver operating characteristic (ROC) curves were constructed and area under the curve (AUC) calculated. Statistical Package for Social Sciences (SPSS) version 20 for windows was used for statistical analysis.

## Results

The mean age of the participants was 46.7 ± 11.2 years. Type 2 DM (T2DM) was present in 91.9 % of the participants and 8.1 % had type 1 DM. Male to female ratio was 1.01. Duration of DM was 6.97 ± 7.1 years. Body mass index was 30.9 ± 7.1 kg/m^2^. History of FU was present in (11) 3.7 % of the participants. Hypertension and dyslipidemia were present in (109) 36.8 % and (144) 48.6 % of the participants respectively and 80.1 % led a sedentary life-style. Diabetic retinopathy was present in (16) 5.4 % of the participants.

Painful neuropathic symptoms (PNS) were present in (154) 52 % of the participants and moderate PNS as defined by NSS ≥ 5 were present in 53 (18.2 %). DPN as defined by NDS ≥ 3 was present in (67) 22.6 % of the participants and 15.9 % had increased risk of FU as defined by NDS ≥ 6. DPN of moderate severity was present in 35 (11.8 %) of the participants.

Quantitative sudorimetry using hands and feet-ESC was performed in all patients. As hands-ESC correlated significantly with feet-ESC (Table 1), we used only feet-ESC to analyze the relationship of sudorimetry with the other variables. Among all participants, 137 (46.3 %) had no SMD: feet-ESC ≥ 70 μS*,* 84 (28.4 %) had moderate SMD: feet-ESC < 70- ≥ 50 μS and 75 (25.3 %) had severe SMD: feet-ESC < 50 μS*.*

**Table 1:** Pearson’s correlation coefficient of feet-ESC with tests of peripheral nerve function among the participants

No	Tests	CC	P
1	ESC-hands	0.592	<0 .0001
2	Pain sensitivity	0.406	<0 .0001
3	Differences in temperature perception	0.304	<0 .0001
4	Achilles Reflex	-0.405	<0 .0001
5	Vibration perception by 128Hz tuning fork	-0.391	<0 .0001
7	Pressure perception by 10 g-MF	-0.300	<0 .0001
8	QST by neurothesiometer:VPT ≥ 25 V	-0.383	<0 .0001
9	Sural nerve amplitude	0.163	<0.014
10	Sural nerve velocity	0.249	<0.0001
11	Peroneal nerve amplitude	0.184	<0.006
12	Peroneal nerve velocity	0.278	<0.0001
13	Painful DPN by NSS ≥ 5	-0.230	<0 .0001
14	DPN by NDS ≥ 3	-0.469	<0 .0001
15	Risk of foot ulcer by NDS ≥ 6	-0.398	<0 .0001
16	Severity of DPN by NDS ≥ 3	-0.442	<0 .0001

*DPN* diabetic peripheral neuropathy. *ESC* electrochemical skin conductance. *NSS* Neuropathy symptom score. *NDS* neuropathy disability score. *QST* quantitative sensory testing. *VPT* vibration perception threshold. 10-g MF 10 gram monofilament. *CC* correlation coefficient

There was a significant negative correlation between feet-ESC and age, duration of DM and a past history of FU (-0.234 *p* < 0 .0001), (-0.301 *p* < 0 .003), and (-0.366 *p* < 0 .0001) respectively. Feet-ESC decreased in the presence of diabetic retinopathy (-0.170 *p* < 0 .003) and elevation of systolic blood pressure (-0.129, *p* < 0 .036). Feet-ESC also decreased with rising levels of serum creatinine (-0.143^,^ *p* < 0 .014) and uric acid (-0.162, *p* < 0 .008). Notably feet-ESC increased with increasing levels of physical activity (0.171, *p* < 0 .0001). Analysis of the relationship between feet-ESC and the levels of fasting blood glucose (FBG) and HbA1c showed an insignificant inverse relationship: (-0.026, *p* < 0.66), and (-0.062, *p* < 0.31) respectively. However there was a positive correlation between both FBG and HbA1c and patients who are in the category of severe SMD (feet-ESC < 50 μS) as a group, (0.118, *p* < 0.044), (0.134, *p* < 0.029) respectively.

Table 1 presents trends in the correlation coefficients between feet-ESC and tests of peripheral nerve function. The values of feet-ESC decreased as the bedside sensory and motor function tests and VPT assessed quantitatively by neurothesiometer increased. The values of feet-ESC also decreased with increasing scores of NSS, NDS ≥ 3, and NDS ≥6 reflecting the presence of PNS, DPN and the risk of foot ulceration (RFU). As the severity of DPN increased, the values of feet-ESC decreased. ESC-feet decreased as the amplitude and conduction velocity of the sensory and motor nerves decreased. Notably there was a positive correlation between feet-ESC and both pain sensation and differences in temperature sensation.

Table 2 presents variances in the mean feet-ESC between participants with and without PNS, DPN, increased risk of FU and confirmed DPN. There was a significant decrease in feet-ESC among participants with DPN compared with those without DPN. Further analysis showed significantly lower values of feet-ESC in patients with confirmed DPN as compared with participants with subclinical DPN (54.7 ± 24.8 vs 70.2 ± 14.14 μS; *P* < 0.0001).

**Table 2:** Variances in feet-ESC between participants without or with painful neuropathic symptoms, diabetic peripheral neuropathy, RFU, and confirmed neuropathy

	NSS ≥ 5	NDS ≥ 3	NDS ≥ 6	Confirmed-DPN
No	67.1 ± 18.4 μS	67.4 ± 16.9 μS	65.6 ± 18.8 μS	67.6 ± 16.9 μS
Yes	57.1 ± 23.7 μS	42.9 ± 26.1 μS	41.9 ± 25.9 μS	53.7 ± 24.7 μS
*P*	<0.0001	<0.0001	<0.0001	<0.0001

*DPN* diabetic peripheral neuropathy, *NSS* Neuropathy symptom score. *NDS* neuropathy disability score. *RFU* risk of foot ulceration

**Fig. 1:**
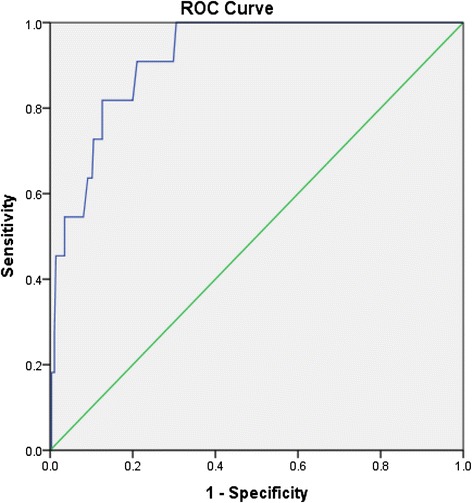
Feet-ESC receiver-operating characteristic curve ROC to reflect diabetic peripheral neuropathy assessed by VPT ≥ 25 V. Area under the curve = 0.918, *P* < 0.0001

Table 3 demonstrates the performance of severe SMD (feet-ESC <50μS) to detect DPN and the risk of foot ulceration, against the traditional tests, in particular: VPT ≥ 25 V, NDS ≥ 3 and NDS ≥ 6. Feet-ESC < 50 μS was sensitive and specific in detecting DPN and the risk of FU among participants. Similarly, feet-ECS <70μS was highly sensitive but less specific in detecting DPN assessed by VPT, NDS and confirmed DPN (defined by the criteria of the American Academy of Neurology 2005 [13]) with a notable high positive predictive (PPV) value for confirmed DPN. Figure 1 demonstrates the ROC curve of feet-ESC to reflect DPN assessed by VPT ≥ 25 V.

**Table 3:** Performance of feet-ESC to detect diabetic peripheral neuropathy and the RFU against traditional tests

	Feet-ESC < 50μS						
	Sensitivity	Specificity	AUC	PPV	NPV	+LH	-LH
DPN:VPT ≥ 25v	90.1	77	0.841	13	99	3.98	0.12
DPN:NDS ≥ 3	61.2	85.2	0.732	54	85	4.12	0.46
RFU:NDS ≥ 6	63.8	81.9	0.729	40	92	3.53	0.44
Confirmed-DPN	38.4	91	0.648	85.7	52	4.3	0.67
	Feet-ESC <70μS						
DPN:VPT ≥ 25v	100	46	0.732	6.7	100	1.86	0
DPN:NDS ≥ 3	80.6	51	0.663	32.5	90	1.66	0.38
RFU:NDS ≥ 6	80.9	49	0.651	23	93	1.59	0.39
Confirmed-DPN	67.2	58.9	0.63	93	56	1.6	0.56

*DPN* diabetic peripheral neuropathy. *RFU* risk of foot ulceration. *ESC* electrochemical skin conductance. *VPT* vibration perception threshold. *NDS* neuropathy disability score. *AUC* area under the curve. *PPV* positive predictive value. *NPV* negative predictive value. *+LH* positive likelihood. *–LH* negative likelihood

## Discussion

We demonstrated that in Saudi patients with DM, severe SMD as defined by a feet-ESC threshold below 50 μS was sensitive and highly specific in detecting DPN assessed by VPT and NDS. Feet-ESC < 50 μS was sensitive and specific in detecting the risk of FU as well.

Furthermore feet-ESC threshold below 70μS, showed moderate sensitivity to detect confirmed-DPN assessed by neurophysiological studies and classified according to the American Academy of Neurology 2005 criteria. In an earlier publication Casellini C et al showed that feet-ESC exhibited high sensitivity and specificity to detect DPN evaluated by the Neuropathy Impairment Score-Lower Legs (NIS-LL) [14]. In patients with type 1 DM, Selvarajah D et al have demonstrated that feet-ESC was sensitive and specific to identify confirmed-DPN classified according to AAN criteria [19].

Additionally we showed that feet-ESC was able to differentiate between participants with and without DPN assessed by NDS and neurophysiological testing. Our results are in agreement with results reported in other studies. [14, 19, 20] These observations indicate that sudorimetry using feet-ESC relates significantly to tests of sensory and motor nerve function that are used to detect large fiber-sensory polyneuropathy (LFSN).

On the other hand we have shown that 53.7 % of the participants had either moderate or severe SMD assessed by feet-ESC. Feet-ESC positively correlated with tests to detect pain sensitivity and differences in temperature sensation, both of which are bedside tests, used to assess small fiber nerve function. Although assessment of symptoms and even quantitative sensory tests for cold, warm and pain sensitivity lack precision in detecting small fiber nerve function [6] Smith et al have shown similar performance of feet-ESC compared to intraepidermal nerve fiber density (IENFD) obtained from skin biopsy that is considered the gold standard for diagnosing small fiber sensory neuropathy (SFSN) [15].

Together these results do not imply that ESC identifies all forms of DPN, but rather reflect its ability to detect the involvement of small C-fiber nerve dysfunction in a generalized DPN in addition to the concurrent occurrence of autonomic and somatic C-fiber dysfunction in SFSN. This fact confers a potential role for ESC to screen for both LFSN and SFSN in addition to SMD. Being simple, non-invasive, quick, and objective and requiring no training or special patient preparation qualifies ESC as a suitable measurement tool to screen for DPN in busy diabetes clinics.

The strength of our study lies in the fact that we performed neurophysiological tests on a large number of participants (222) to define DPN in addition to bedside tests.

We also demonstrated that in Saudi patients with DM, quantitative sudorimetry using feet-ESC was related not only to traditional tests for identifying DPN but also to the factors that are associated with increased risk for developing DPN. Feet-ESC decreased with increasing age, duration of DM, systolic blood pressure and a past history of FU. Earlier Yajnik et al demonstrated a positive correlation of feet-ESC with age and duration of DM [20]. A decrease in the feet-ESC levels was observed with rising levels of serum uric acid. This is in agreement with results reported by Papanas N et al who demonstrated that increased levels of uric acid represents a risk factor for DPN in general and SMD in particular [21, 22].

Analysis of the relationship between indices of glycemic control and feet-ESC showed a significant positive correlation between both fasting blood glucose levels, HbA1c and feet-ESC threshold of severe SMD (feet-ESC < 50 μS) that probably represents established SMD. Results are in agreement with findings by Yajnik et al, [20] and imply that uncontrolled DM increases the risk of developing SMD. Decreased feet-ESC was also associated with the presence of diabetic retinopathy and increasing levels of serum creatinine. Gin et al also demonstrated an inverse relationship between ESC and diabetic retinopathy (-0.42, *p* < 0.0001) [23]. The relationship demonstrated in our study between indices of blood glucose control and severe SMD ie (feet-ESC < 50 μS), the presence of diabetic retinopathy and increasing levels of creatinine (the three of which represent indicators of diabetic microvascular complications) is in agreement with the results of the landmark studies that have shown a direct effect of glycemic control on the development of diabetic microvascular complications including DPN that encompasses SMD as well [24, 25]. It is worth mentioning that several recent studies have suggested utilization of a risk score based on ESC as a tool to screen for chronic kidney disease and microvascular complications [16, 26, 27].

An important finding of the study is the relationship between feet-ESC and the level of physical activity among the participants. Participants with a sedentary life-style were at increased risk of SMD reflected by decreased feet-ESC. This result emphasizes the role of PA in protecting sudomotor small C-fiber nerves and is in agreement with results reported by Raisanen A et al who demonstrated a significant increase in estimated VO2max and hands and feet-ESC observed after lifestyle intervention [28].

We did not compare sudorimetry using ESC with other traditional tests to detect autonomic dysfunction. This might be considered a weakness of our study; however others have shown that ESC significantly correlated with the quantitative autonomic testing and with the quantitative sudomotor axon reflex testing [14, 15].

Our study has demonstrated that more than half of the studied Saudi patients with DM newly referred to a specialized diabetes center had SMD assessed quantitatively using ESC and could thus be at risk of developing FU. In this regard it is worth mentioning that a recent cohort study has demonstrated that the prevalence of diabetic foot complications among Saudi Arabian patients with DM is 3.3 %, and is within the estimated international range [29]. This burden is expected to grow as the prevalence of DM continues to rise in Saudi Arabia [8]. Simplicity and reliability are therefore prerequisites for a screening method to identify patients at risk of FU, to be widely utilized as the prevalence of DM continues to rise. The study showed that measuring ESC is a simple, noninvasive, reliable, quantitative test that can be introduced at a point of care to screen for SMD, DPN and the risk of FU. Utilization of ESC will simplify screening for DPN to identify patients at risk who are in need of special care to prevent limb loss. Further studies are warranted to support the results and prospective studies should explore the potential of ESC to predict the development of FU and test the effectiveness of interventions to prevent SMD and FU.

## Conclusion

Our study demonstrated that measuring feet-ESC is a simple, noninvasive method with sufficient sensitivity and specificity to identify patients with DM who are at risk of FU.

## Abbreviations

DM, diabetes mellitus; DPN, diabetic peripheral neuropathy; ESC, electrochemical skin conductance; FU, foot ulceration; NDS, neuropathy disability score; NSS, neuropathy symptom score; PNS, painful neuropathic symptoms; QST, quantitative sensory testing; SMD, sudomotor dysfunction; VPT, vibration perception threshold
